# Characterization of the Proteins Secreted by Equine Muscle-Derived Mesenchymal Stem Cells Exposed to Cartilage Explants in Osteoarthritis Model

**DOI:** 10.1007/s12015-022-10463-4

**Published:** 2022-10-22

**Authors:** Lola Dechêne, Margaux Colin, Catherine Demazy, Maude Fransolet, Ariane Niesten, Thierry Arnould, Didier Serteyn, Marc Dieu, Patricia Renard

**Affiliations:** 1 Laboratory of Biochemistry and Cell Biology (URBC), NARILIS (Namur Research Institute for Life Sciences), University of Namur (UNamur), Rue de Bruxelles 61, 5000 Namur, Belgium; 2 Department of Clinical Sciences, Anesthesiology and Equine Surgery, Faculty of Veterinary Medicine, B41, University of Liege, Sart Tilman, 4000 Liège, Belgium; 3 Department of Pharmacotherapy and Pharmaceuticals, Faculty of Pharmacy, Université Libre de Bruxelles (ULB), 1050 Brussels, Belgium; 4 Mass Spectrometry Platform (MaSUN) - Namur Research Institute for Life Sciences (Narilis), University of Namur (UNamur), 5000 Namur, Belgium; 5 Centre of Oxygen, Research and Development (CORD), Institute of Chemistry B6a, University of Liege (ULiège), Sart Tilman, 4000 Liège, Belgium

**Keywords:** Equine mesenchymal stem cells, Secreted proteins, Osteoarthritis, Decorin, Matrix metalloproteinase 3 (mmp3), SILAC, Cartilage explant

## Abstract

**Background:**

Osteoarthritis (OA) is a highly prevalent joint degenerative disease for which therapeutic treatments are limited or invasive. Cell therapy based on mesenchymal stem/stromal cells (MSCs) is therefore seen as a promising approach for this disease, in both human and horses. As the regenerative potential of MSCs is mainly conferred by paracrine function, the goal of this study was to characterize the secreted proteins of muscle-derived MSCs (mdMSCs) in an in vitro model of OA to evaluate the putative clinical interest of mdMSCs as cell therapy for joint diseases like osteoarthritis.

**Methods:**

An equine osteoarthritis model composed of cartilage explants exposed to pro-inflammatory cytokines was first developed. Then, the effects of mdMSC co-culture on cartilage explant were studied by measuring the glycosaminoglycan release and the NO_2_^−^ production. To identify the underlying molecular actors, stable isotope-labeling by amino acids in cell culture based secreted protein analyses were conducted, in the presence of serum. The relative abundance of highly sequenced proteins was finally confirmed by western blot.

**Results:**

Co-culture with muscle-derived MSCs decreases the cytokine-induced glycosaminoglycan release by cartilage explants, suggesting a protecting effect of mdMSCs. Among the 52 equine proteins sequenced in the co-culture conditioned medium, the abundance of decorin and matrix metalloproteinase 3 was significantly modified, as confirmed by western blot analyses.

**Conclusions:**

These results suggest that muscle-derived MSCs could reduce the catabolic effect of TNFα and IL-1β on cartilage explant by decreasing the secretion and activity of matrix metalloproteinase 3 and increasing the decorin secretion.

**Graphical abstract:**

mdMSCs capacity to reduce the catabolic consequences of cartilage exposure to pro-inflammatory cytokines. These effects can be explained by mdMSC-secreted bioactive such as TIMP-1 and decorin, known as an inhibitor of MMP3 and an anti-inflammatory protein, respectively. 
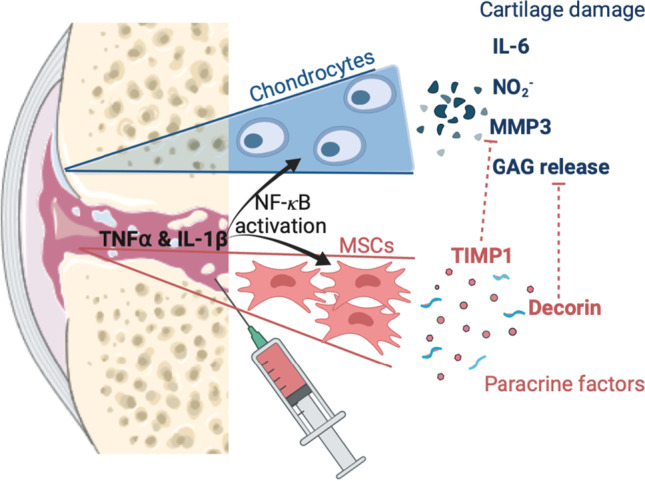

**Supplementary Information:**

The online version contains supplementary material available at 10.1007/s12015-022-10463-4.

## Background

Osteoarthritis (OA) is a degenerative disease that concerns over 10% of people over 60 years of age worldwide. OA etiology includes genetics, metabolic factors, inflammation and trauma [1]. OA is a chronic condition that leads to irreversible tissue degradation due to inflammation [2]. Lesions are multiple and concern all constituents of the joint: cartilage degradation, synovial inflammation and bone lesions (osteophytes, subchondral sclerosis and bone marrow lesions) [3]. Mostly administered therapies are symptomatic with administration of anti-inflammatory drugs, although surgery (joint replacement) is the only option available for joints with high grade of OA. The main challenge is to identify therapeutic strategies decreasing the progression of the disease and treating existing lesions [4]. Therefore, regenerative therapies based on stem cells are promising treatments for joints suffering from OA and several clinical trials showed that stem cell therapy can be a reliable treatment for this disease [1], with improvement of clinical outcomes [5]. In addition, treating OA with intra-articular injection of mesenchymal stem/stromal cells (MSCs) has been described to decrease joint inflammation and lead to cartilage regeneration in several animal species (such as mice, rats, pigs, horses or donkeys) [6]. Therapeutic properties result from the capacity of MSCs to reach injured tissues, to secrete trophic factors to allow regeneration and to modulate the inflammatory environment of OA [7].

The main tissue sources of MSCs are primarily the umbilical cord for neonatal tissue and bone marrow or adipose tissue for adult tissues [8]. However, MSCs can be harvested from many other vascularized tissues such as peripheral blood, lung, synovial fluids, periodontal ligaments or muscles [9]. In this study, we investigated the potential of muscle-derived MSCs (mdMSCs) as skeletal muscle has the advantage to represent more than 40% of horse body mass and is easily accessible [10, 11]. mdMSCs are thus an attractive medical therapy according their relative ease of isolation and purification [12].

In this study, we explored the potential of equine mdMSCs as a potential therapy for OA for two main reasons. The first one is that the horse is a relevant model for human OA. Indeed, the horse is an athletic species with a relatively long lifespan similar to human [13]. Horses are also considered being the most anatomically similar animal model to humans to study articular therapy before clinical trials [14]. Horse joints share a lot of common features with human joints as articular cartilage thickness [15]. Cellular structure and biochemical composition of horse cartilage are two other features very comparable to human cartilage [16] and horse stifle is described to be similar to human knee [17]. More importantly, OA prevalence is naturally high in this species [18] because racehorses are submitted to repetitive impacts and therefore to joint injuries during their athletic career [19]. A lot of data elements are already published for this species such as clinical tests, imaging and rehabilitation techniques for horses suffering from osteoarthritis [20], making OA a well-known and characterized disease in horse. Considering horse as an animal model for human OA also provides potential access to moderately damaged tissues, on the contrary to human tissues that are often only available after joint replacement at the end stage of the disease when little intervention can be concluded [21]. The second reason is that, as horse naturally suffers from OA, mdMSCs might eventually represent a suitable therapeutic approach for high value horses such as racehorses. Indeed, the growing use of MSCs in the context of horse joint diseases such as OA or tendinitis [22, 23] has shown their potency to reduce pain and cartilage damages (reviewed in Voga et al. [24]). MSCs represent a high therapeutic potential in veterinary medicine for several species [25, 26]. However, numerous factors remain to be determined to define the best MSCs-based OA therapy, such as the dose, the time of implantation, the tissue of origin, the health status of the donor [27], the number of injections required [28], the use of allogenic or autologous MSCs [29, 30], or the combination with a complementary therapeutic approach [24].

The in vitro equine osteoarthritis model developed in this work consists in explants of cartilage exposed to inflammatory cytokines, more precisely tumour necrosis factor (TNF)α and Interleukin (IL)-1β, as this was described as the best method to mimic OA-induced cartilage damage [17]. TNFα and IL-1β exposure induces the release of glycosaminoglycans (GAG), nitrite (NO_2_^−^) and IL-6 in the supernatant of cartilage plugs. We showed that the GAG release is less pronounced when cartilage plugs are co-cultured with mdMSCs.

The therapeutic effect of MSCs is largely attributed to their paracrine activity [9]. The factors secreted by MSCs and contributing to their immunomodulatory potential are also largely influenced by their microenvironment [31]. For example, the secretome of human MSCs cultured with pro-inflammatory cytokines (IL-1β, IL-6 and TNFα) showed an up-regulation of proteases (Matrix Metalloproteinase (MMP) 1, MMP2, MMP3,…) and inhibitors of proteases (like Serine proteinase inhibitors (SERPIN) E1 and Tissue inhibitor of metalloproteinases (TIMP) 1) compared to the secretome of non-treated MSCs [32]. The presence of serum in the culture media also influences the secretome and leads to increase the amount and the variety of paracrine factors identified in the supernatant of MSCs when compared with factors recovered from serum-starved cells [33].

However, secreted protein analysis of cells cultured in presence of fetal bovine serum (FBS) represents a technical challenge due to the presence of highly abundant FBS proteins (such as bovine serum albumin or immunoglobulins) that increase the complexity and the dynamic range of the supernatant samples. More precisely, the challenge is due to the low amount of proteins secreted by cells when compared to the high amount of serum proteins in cell culture media for in vitro analyses [34]. Although serum deprivation decreases sample complexity and increases reproducibility [34], serum starvation leads to both decrease the amount of proteins secreted by MSCs and to modify the secretome profile [33].

We therefore chose to profile secreted proteins of mdMSCs in the presence of serum, but using a specific proteomic method based on a metabolic labelling strategy with stable isotope-labeling by amino acids in cell cultures (SILAC) [35]. During several passages, cells incorporate labeled amino acids—more precisely medium or heavy isotopes of amino acids—placed in the culture media to synthetize labeled proteins [34]. Once the incorporation level is sufficient, collected supernatants are analyzed by mass spectrometry that allows to distinguish labelled peptides derived from mdMSCs secreted proteins from FBS-derived unlabeled peptides. SILAC therefore offers an alternative way to distinguish secreted proteins from bovine contaminant proteins. In this study, we applied, for the first time, the SILAC method to study secreted proteins by mdMSCs in an in vitro OA model.

Altogether, these data characterize the paracrine property of mdMSCs in an in vitro osteoarthritis model and supports mdMSCs as a valuable source of MSCs in OA cell therapy.

## Materials and Methods

### Muscle-Derived Mesenchymal Stem Cells

The equine skeletal mdMSCs were provided by RevaTis (Aye, Belgium). They were cultured in Dulbecco’s modified Eagle’s medium (DMEM) F12 culture medium (Gibco) supplemented with 20% heat-inactivated FBS (Gibco), 100 IU/mL of penicillin–streptomycin (Gibco) and 0.5% of amphotericin B (Gibco) at 37 °C and 5% CO_2_ according to the recommendations of RevaTis [10]. Cells were used between passages 4 and 6.

TNFα (1 ng/mL) (Bio-Techne, 210-TA) and IL-1β (0.1 ng/mL) (Bio-Techne, 201-LB) added to cell culture medium (DMEM F12 with 20% of FBS) constitute the hereafter called “inflammatory medium”.

### Cartilage Sample Collection

Plugs of cartilage were harvested from horses euthanized for other reasons than osteoarthritis disease and after obtaining owners’ consent. Briefly, cartilage was obtained from stifle joint, with a scalpel blade (size 20), on femoral trochlea through a sterile access to the joint caudal to the lateral patellar ligament. Plugs of cartilage were weighed and placed in the same culture medium as mdMSCs during 3 days before their use for experiments. Cartilage explants were kept in culture for a maximum of 15 days.

### Chondrocytes Viability Assessment

Thin slices of cartilage were cut and then incubated with 2 mL of PBS – Ethidium Bromide (final concentration of 10 μg/mL) (Sigma-Aldrich) and Acridine Orange (final concentration of 3 μg/mL) solution in 2-well Chambered Coverglass Nunc Lab-Tek (Thermofisher) for 30 min at 37 °C and protected from light. Then, the fluorescence intensities of red and green emission signals (wavelengths 500-570 nm and 615-675 nm, respectively) were measured with confocal microscopy (SP5, Leica) by using Z stacking.

### Nuclear Translocation of NF-kB in mdMSCs (Western Blot Analysis on Nuclear Fractions)

Nuclear fractions from mdMSCs were prepared as described by Dignam et al. [36]. Briefly, cells were washed twice with cold PBS, scrapped and centrifuged for 10 min at 200 g and 4 °C. Lysis buffer (20 mM HEPES; pH 7.5, 0.35 M NaCl, 20% glycerol, 1% NP-40, 1 mM MgCl_2_·6H_2_O, 0.5 mM EDTA, 0.1 mM EGTA) was added to the pellet for 10 min on ice before additional centrifugation (20 min at 18,000 g and 4 °C). The supernatant containing nuclear proteins was harvested and protein concentration was determined with Pierce assay (Thermofisher). For western blot analysis, 5 μg of proteins were incubated at 95 °C for 5 min and then resolved by sodium dodecyl sulfate (SDS) polyacrylamide gel electrophoresis on a 12% gel, transferred to a polyvinylidene fluoride membrane (ThermoFisher). The membrane was blocked for 1 h at room temperature (RT) with Odyssey Blocking buffer (Li-Cor Biosciences) followed by overnight incubation with the following primary antibodies diluted 1000 times in blocking buffer with 0.1% of Tween-20 (Bio-Rad Laboratories): anti-p65 (Cell Signaling # CSD14E12) or anti-TATA box binding protein (TBP/TFIID) (Santa Cruz # SC204). After 3 washes with PBS-2% Tween-20, the incubation with the secondary antibody (anti-rabbit IgG, Li-Cor Biosciences R700, #926–68,071) diluted 10 000 times was performed for 1 h followed by 3 washes with PBS- 2% Tween-20. The membrane was scanned with odyssey Infrared Imager (Li-Cor Biosciences) and fluorescence intensity was quantified with the Odyssey V3.0 software (Li-Cor Biosciences). TBP immuno-detection was used as a loading control.

### RT-qPCR

Total RNA was extracted with ReliaPrep RNA Miniprep Systems (Promega) as recommended by the manufacturer. An amount of 2 μg of total RNA was reverse transcribed using GoScript Reverse Transcription mix Oligo(dT) (Promega) following supplier’s instructions. GoTaq qPCR Master Mix (Promega) was used to perform amplification with a Viia7 equipment (Applied Biosystems). Primers (listed in supplementary information, additional file 1, Table 1) used at a concentration of 300 nM. All the results were normalized to the mRNA abundance of enolase using the 2^−∆∆Ct^ method and expressed as fold changes of the untreated condition. Data was analyzed using the Kruskal–Wallis test followed by the Dunn’s multiple comparison.

### Phenotypic Analysis of Cartilage Plugs: Response to Inflammatory Cytokines

Supernatants of cartilage plugs were collected after 3 days of culture in pro-inflammatory culture media and abundances of nitrite, glycosaminoglycan and IL-6 were quantified.

- **Nitrite dosage**: After the centrifugation of the supernatant (5 min, 200 g, at RT), 50 μL of each sample were used to quantify NO_2_^−^ with the Griess system reagent (Promega). Nitrite dosage was performed in duplicate according to the manufacturer’s instructions. Absorbance was measured at 535 nm with a spectrophotometer (xMark, Bio-Rad). Results were normalized for the mass of plugs.
- **Glycosaminoglycan release dosage**: Blyscan sulfated glycosaminoglycan assay (Biocolor) was performed on 10 μL of supernatant according to the manufacturer’s recommendations. Results were normalized to the mass of the plugs.
- **IL-6 ELISA**: The levels of secreted IL-6 in cell culture supernatant were assessed using an Equine IL-6 DuoSet ELISA (R&D Systems, United Kingdom) according to manufacturer’s instructions. Results were normalized on the mass of the plugs.

### Clusterin, Decorin and MMP3 Abundance (Western Blot Analyses)

Supernatants were harvested, centrifuged (5 min, 200 g and RT) to remove cell debris and then protein concentrations were measured with Pierce assay (Thermofisher). An amount of 35 μg of proteins were resolved by western blot as described in the part “Nuclear translocation of NF-kB”. Primary antibodies used were diluted 1000 times (anti-clusterin antibody, Aviva Systems Biology #ARP61142; anti-decorin antibody, ThermoFisher #PA527370) or 750 times (anti-MMP3 antibody, Aviva Systems Biology #ARP42042). The membrane was scanned with Typhoon IR (Cytiva) and fluorescence was quantified with the ImageQuant software (Cytiva). Red ponceau staining was used as a loading control.

### MMP3 Activity

MMP3 activity was monitored thanks to the fluorescence generated by the cleavage of a synthetic substrate (Sigma, MAK291). Briefly, 50 μl of cell supernatant was incubated for one hour with 50 μl of substrate diluted in the assay buffer. The fluorescence resulting from MMP3 activity was measured with Spectramax I3 (Molecular Devices) and compared between conditions.

### Co-Culture of Cartilage Explants and mdMSCs

Approximately 230 mg of cartilage plugs were primed with 1 ng/mL TNFα and 0.1 ng/mL IL-1β for 3 days in 12 well-culture plates (Corning) with 4 mL of inflammatory media per well before being placed in 25 cm^2^ culture flasks (Corning) containing mdMSCs at 80 % confluency, still in the presence of the pro-inflammatory cytokines. After 1, 4 and 9 days of co-cultures, supernatants were collected to assess GAG release and NO_2_^-^ production.

### Secreted Protein Analysis

#### Labelling: SILAC Adaptation Phase

For SILAC adaption phase, cells were cultured with DMEM F12 for SILAC (medium deprived of arginine and lysine, Thermofisher, 88370) supplemented with 20 % of dialyzed FBS (Thermofisher, A3382001), HEPES (15 mM), 100 IU/mL of penicillin-streptomycin, 0.5 % of amphotericin B and stable isotopes listed in supplementary information (additional file 1, Table 2). The use of dialyzed serum is recommended to avoid the uptake of non-labelled amino acids by the cells [37]. To prevent arginine to proline conversion, L-Proline (200 mg/L) was added to the medium [38].

Incorporation of stable isotopes was assessed by detecting and quantifying the unmodified and the stable isotope-modified forms of peptides. Such analysis is performed by the so-called post-translational modifications (PTM) profile tool analysis of the PEAKS Studio X Pro (Bioinformatics Solutions Inc., Waterloo, ON). Practically, a comparative analysis of cell lysates from cells treated with TNFα and IL-1β compared with non-treated cells was performed. The first 10 Equus proteins (represented by 413 peptides) with the most unique peptides in the proteome were examined with the PTM profile tool. Sums of extracted ion current areas corresponding to modified and to unmodified forms of each of these peptides were measured. Then, the incorporation percentage was calculated with the following formula: total area of the modified form / (total area of the modified form + total area of the unmodified form).

#### SILAC Experimental Phase

mdMSCs isotope-labelled for 2 passages were cultured in 25 cm2 flask to 80% confluency. The medium was replaced by inflammatory medium added with approximately 230 mg of plugs of cartilage. After 24 h, plugs of cartilage, mdMSCs and supernatants were harvested. As SILAC experimental design is recommended to include forward and reverse label-swap to limit the possible errors in quantification [39], the same experiments were performed with mdMSCs at passages 4 and 5 but with swapped labelling. For instance, mdMSCs challenged with pro-inflammatory cytokines were collected at passage 4 with the medium labelling and at passage 5 with the heavy labelling. Thus, each experimental condition was obtained for both heavy and medium labels.

- After centrifugation (5 min, 200 g at RT) to eliminate cellular debris, supernatants were concentrated (concentration factor: ± 10x) using Amicon Ultra-4 centrifugal filters ultracel-3 K during 40 min at 4 °C and 2600 g. Then, the protein content of supernatants was measured with Pierce assay (Thermo Fisher, 22,660). An amount of 15 μg of concentrated supernatant of a medium-labelled condition were mixed with 15 μg of heavy-labelled experimental condition. The mix was immediately digested with trypsin and the remaining volumes of supernatants were fractionated and frozen at -80 °C. Digestion was performed as described below.
- Cells were washed with PBS and detached with scrapper in lysis buffer (20 mM Tris–HCl; pH 7.5, 150 mM NaCl, 15% Glycerol, 2% SDS, complete protease inhibitor cocktail (Roche 11,697,498,001), phosphatase inhibitors (1 mM of Na_3_VO_3_, 10 mM 4-nitrophenylphosphate, 10 mM β-glycerophosphate and 5 mM NaF, final concentrations), 1%, Triton X-100 containing 1% SuperNuclease (Sino Biologicals, 25 U/μL)) before frozen at -80 °C.

#### Mass Spectrometry Analysis 

After extraction of proteins in buffer containing 7 M urea, 2 M thiourea, 1% CHAPS, 1% ASB14, 30 mM Tris, 1% SDS at pH 8.5, protein concentration was determined with Pierce assay (Thermo Fisher, 22,660) and 35 μg were processed. Digestion was performed with a modified filtered-aided sample preparation [40] that consists in diluting samples in urea buffer (0.1 M Tris–HCl, 8 M Urea, pH 8.5), reducing proteins with 8 mM dithiothreitol, alkylating them with 50 mM iodoacetamide and digest protein with trypsin (Promega) (1:50 w:w) on Microcon Ultracel PL-30 (Millipore). After final concentration of the samples with speed vac, samples were suspended in 2% acetonitrile and 0.1% trifluoroacetic acid (final concentration 500 ng/μL) before being frozen at -80 °C until mass spectrometry (MS) analysis.

The digest was analyzed using nano-LC–ESI–MS/MS tims TOF Pro (Bruker, Billerica, MA, USA) coupled with an UHPLC nanoElute (Bruker). Peptides were separated by nanoUHPLC (nanoElute, Bruker) on a 75 μm diameter, 25 cm C18 column with integrated CaptiveSpray insert (Aurora, ionopticks, Melbourne) at a flow rate of 400 nL/minutes, at 50 °C. LC mobile phase A was water with 0.1% formic acid (v/v) and phase B was acetonitrile with 0.1% formic acid (v/v). Samples were directly loaded on the analytical column at a constant pressure of 800 bar. The digest (1 μL) was injected, and the organic content of the mobile phase was increased linearly from 2% B to 15% in 36 min, from 15% B to 25% in 19 min, from 25% B to 37% in 5 min and from 37% B to 95% in 5 min. Data acquisition on the tims TOF Pro was performed using Hystar 5.1 and timsControl 2.0. tims TOF Pro data were acquired using 100 ms TIMS accumulation time, mobility (1/K0) range from 0.6 to 1.6 Vs/cm^2^. Mass spectrometry analyses were carried out using the parallel accumulation serial fragmentation (PASEF) acquisition method [41]. One MS spectrum was followed by ten PASEF MS/MS spectra per total cycle of 1.1 s. Two injections per sample were done.

Data analysis was performed using PEAKS Studio X Pro with ion mobility module and Q module for SILAC quantification. Protein identifications were conducted using PEAKS search engine with 15 ppm as parent mass error tolerance and 0.05 Da as fragment mass error tolerance. Carbamidomethylation was allowed as fixed modification, oxidation of methionine, 4,4,4,4-D4 Lysine, 13C[6] silac label, 13C[6] 15 N[4] silac label, 13C[6] 135 N[2] silac label as variable modifications. Enzyme specificity was set to trypsin, and the maximum number of missed cleavages per peptide was set to one. The peak lists were searched against the proteome databases of Equus Caballus and Bos Taurus from UNIREF 100 (96,566 sequences). Peptide spectrum matches and protein identifications were normalized to less than 1.0% false discovery rate and with SILAC-3plex quantitation auto normalization method. For the quantitation, mass error and ion mobility tolerance were set respectively to 15 ppm and 0.05 1/k0. For the SILAC quantitation results, feature vector filter quality score was set to ≥ 50 and protein significance score threshold was set to 20. The significance score was calculated as the -10log10 of the significance testing p-value (0.01). Paired t-test was used as the significance testing method. Only proteins with at least two peptides were used for the quantification.

#### Statistics

Statistical analyses were performed with Prism. p values for pro-inflammatory gene expression were calculated using a Kruskal–Wallis test. p values of other assays were calculated with Wilcoxon test. *p* value was considered statistically significant below < 0.5 (* = 0.05 < *p*≧0.01; ** = 0.01 < *p* > 0.001; *** = *p* < 0.001).

## Results

### Set Up and Characterization of an In Vitro Equine Model of Osteoarthritis

Exposing cartilage explants to inflammatory cytokines is regularly used as a relevant model to study osteoarthritis for human and horse disease [42–46]. In order to develop a similar model for equine osteoarthritis, cartilage plugs were first sampled on euthanized horses and evaluated for viability in culture conditions.

**Fig. 1:**
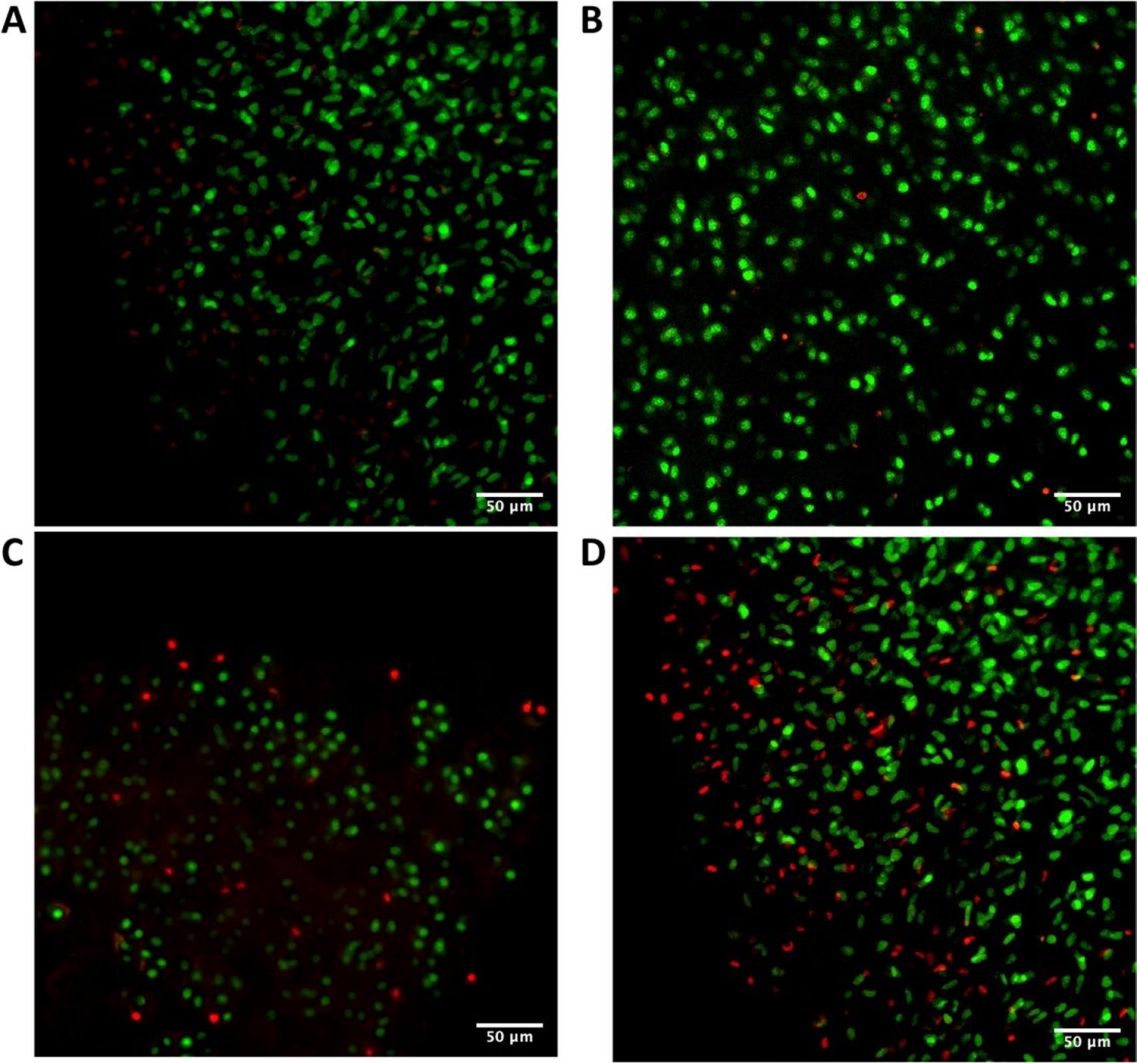
Viability of ex vivo culture of cartilage plugs up to 15 days after sample collection. Fluorescent micrographies of cartilage plugs stained with ethidium bromide (red staining of dead chondrocytes) and acridine orange (green staining of living cells) at 5 (**A**), 9 (**B**), 13 (**C**) and 15 (**D**) days after collection. Cell counting on confocal micrographs revealed that 84% of chondrocytes are still alive 15 days after the collection. Magnification 400x

Ethidium bromide and acridine orange incorporation was used to assess the viability of chondrocytes present in cartilage plugs cultured in vitro for 5 to 15 days (Fig. 1). Viable (green) and dead (red) cells were counted, and 16% of cell mortality were measured after 15 days in culture. These results indicate that chondrocytes are largely kept alive in plugs cultured for up to 15 days after sampling.

**Fig. 2:**
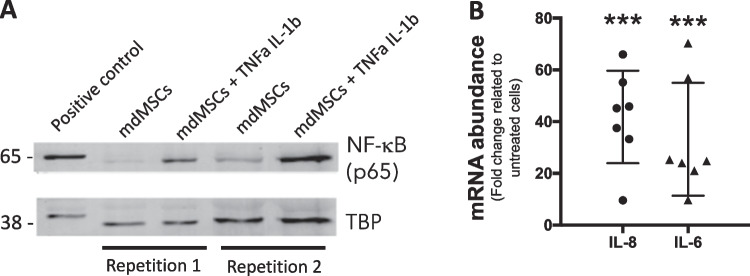
Exposure of equine mdMSCs to human pro-inflammatory cytokines induces the expression of NF-kB-dependent genes. (**A**) Western blot analysis of NF-kB p65 subunit in nuclear fractions of non-treated or treated mdMSCs with TNFα (1 ng/mL) and IL-1β (0.1 ng/mL) for 1 h. Detection of TBP is used as a loading control from the same gel with no cropping in between the bands. Positive control is a nuclear fraction of murine RAW cells treated with 20 ng/mL of LPS for 1 h and previously characterized to assess the nuclear translocation of p65. Full length blots and quantification are included in the Additional file 1 Figs. 1 and 2. (**B**) Gene expression analysis by RT-qPCR of IL-8 and IL-6 in mdMSCs treated for 24 h with TNFα (1 ng/mL) and IL-1β (0.1 ng/mL). The results were normalized to the mRNA abundance of enolase (housekeeping gene) and expressed in fold change related to untreated cells, using the 2.^−∆∆Ct^ method. Each measure is represented by a black dot (IL-8) or a black triangle (IL-6). *p* values were calculated following Kruskal–Wallis test (*n* = 7). *** corresponds to *p*-value < 0.001

After defining the articular part of the in vitro osteoarthritis model, a cocktail of human recombinant TNFα and IL-1β at low concentration was administered to cells as these 2 pro-inflammatory cytokines are present in joints suffering from OA [47]. To test the responsiveness of equine cells to human pro-inflammatory cytokines, we evaluated the activation of NF-kB, a master regulator of inflammatory response. This transcription factor is sequestered in the cytosol in resting cells, and translocates into the nucleus in response to pro-inflammatory signals [48]. Western blot analysis of nuclear fractions of mdMSCs (Fig. 2A) shows the localization of p65, one of NF-kB subunits, in the nuclei of cells exposed to TNFα and IL-1β treatment, indicating that equine cells do respond to low concentrations of these human cytokines. Quantification of this blot (Figure 2 in Additional file) reveals that p65 is 3,6 times more abundant in inflammatory condition compared to non-treated cells. This was confirmed by gene expression analysis of IL-8 and IL-6, two NF-kB-responsive pro-inflammatory genes that are strongly induced by TNFα and IL-1β, used at 1 ng/mL and 0.1 ng/mL, respectively (Fig. 2B).

**Fig. 3:**
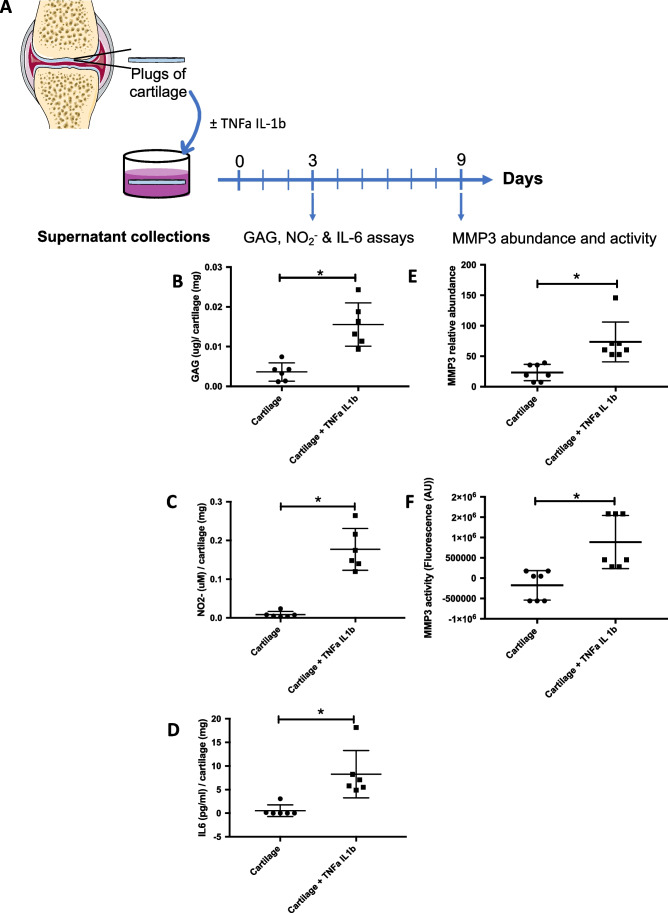
Characterization of the in vitro osteoarthritis model. (**A**) Experimental design: cartilage explants were cultured and exposed to a cocktail of TNFα (1 ng/mL) and IL-1β (0.1 ng/mL) for 3 or 9 days before analyses of supernatants. (B-F) The supernatants were collected after 3 days and analyzed for GAG, NO_2_^−^ and IL-6 content, and after 9 days for MMP3 abundance and activity. The pro-inflammatory phenotype of plugs of cartilage treated with TNFα and IL-1β (black squares) for 3 days displays an increase in the release of GAG in supernatant (**B**), in the formation of NO_2_.^−^ (**C**) and secretion of IL-6 (**D**) compared to non-treated cartilage (black dots). After 9 days, increases are measured in MMP3 abundance (**E**) and activity (**F**). *p* values were calculated following Wilcoxon tests (*n* = 6 for B, C, D; *n* = 7 for E, F); * corresponds to *p*-values < 0.05

Cartilage plugs exposed to this cocktail of TNFα and IL-1β were therefore considered as a simplified in vitro model of osteoarthritis. The response of cartilage plugs to this pro-inflammatory cocktail was further characterized by measuring the abundance of GAG, nitrite, MMP3 and IL-6, in the supernatant [42, 45, 49–52]. A significant increase in these inflammatory markers (Fig. 3B, C and D) was highlighted after 3 days of TNFα and IL-1β exposure. The activity and abundance of MMP3 were also significantly increased for cartilage explants cultured in pro-inflammatory media for an extra 9 days when compared to explants cultured without TNFα and IL-1β (Fig. 3E and F). Altogether, these results indicate that plugs of cartilage cultured with 1 ng/mL TNFα and 0.1 ng/mL IL-1β develop a “OA-like” phenotype and constitute a good and representative osteoarthritis in vitro model for this pathology.

### Protection of mdMSCs toward the GAG Release by Cartilages

**Fig. 4:**
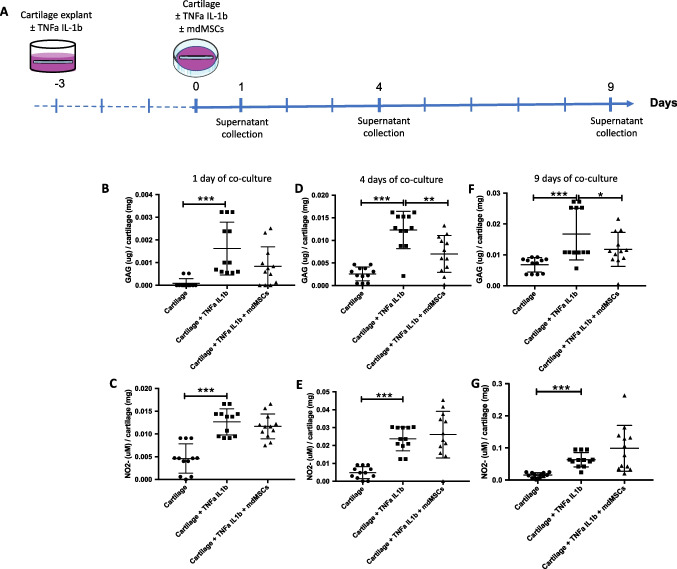
Effect of mdMSCs co-culture on GAG and NO_2_^−^ release by “OA-like” cartilage plugs. (**A**) Experimental design: cartilage explants were primed (or not) for 3 days with pro-inflammatory cytokines to produce “OA-like” cartilage plugs. After this priming, cartilage plugs were exposed (or not) to adherent mdMSCs at 80% of confluency for an additional 9 days, in the presence of pro-inflammatory medium. The supernatants were collected after 1, 4 and 9 days of co-culture to assess GAG release (**B, D, F**) and NO_2_.^−^ production (**C, E, G**) by “OA-like” cartilage plugs. Black dots represent the cartilage plugs not exposed to mdMSCs nor to pro-inflammatory cytokines after priming. Black squares represent the “OA-like” cartilage plugs exposed to inflammatory cytokines but not to mdMSCs co-culture. Black triangles correspond to “OA-like” cartilage plugs exposed to inflammatory cytokines and to mdMSCs co-culture. *p*-values were calculated followed Wilcoxon tests (mdMSCs (*n* = 10), plugs (*n* = 6)). * Corresponds to *p*-value < 0.05; ** corresponds to *p*-value < 0.01; *** corresponds to *p*-value < 0.001

To determine whether the presence of adherent mdMSCs could have a functional effect on the inflammatory phenotype of “OA-like” cartilage or not, we measured the GAG release and the NO_2_^−^ production in the supernatant of flasks containing both adherent mdMSCs and plugs of “OA-like” cartilage, 1, 4 and 9 days after the priming of cartilage (the experimental design is depicted in Fig. 4A). The presence of adherent mdMSCs in flasks significantly decreased the GAG release from primed cartilage plugs in the supernatant after 4 and 9 days of co-culture (Fig. 4B, D and F). NO_2_^−^ produced by cartilage plugs was not influenced by the presence of mdMSCs no matter the time point of the co-culture is (Fig. 4C, E and G).

To analyze the molecular mechanisms underlying this anti-catabolic effect of mdMSCs on cartilage in pro-inflammatory condition, we decided to profile the secreted proteins in this model by proteomic analysis. As explained in the introduction section, a SILAC method was selected in order to avoid serum starvation that could modify the profile of the secreted proteins.

First of all, the experimental conditions of the adaptation phase needed to be defined in order to reach the minimum labelling level, recommended as 97% according to Ong and Mann [35]. The incorporation of stable isotopes was assessed by mass spectrometry analysis. The average obtained for a total of 413 peptides sequenced from 10 equine proteins reached 96% of incorporation after two passages in the presence of the SILAC medium. We considered that this labelling level was sufficient as the incorporation rate depends slightly on the three donors (Table 3 in additional file part) for which incorporation levels were ranging between 94 and 98%. In addition, for 2 donors, the incorporation rates were not different between forward or reverse conditions (names of the 2 technical replicates to obtain each experimental condition in medium and heavy labelling), suggesting that the adaptation phase is sufficient to ensure an almost complete incorporation of labelled amino acids as the reverse experiment is performed at one later passage than forward experiment.

**Fig. 5:**
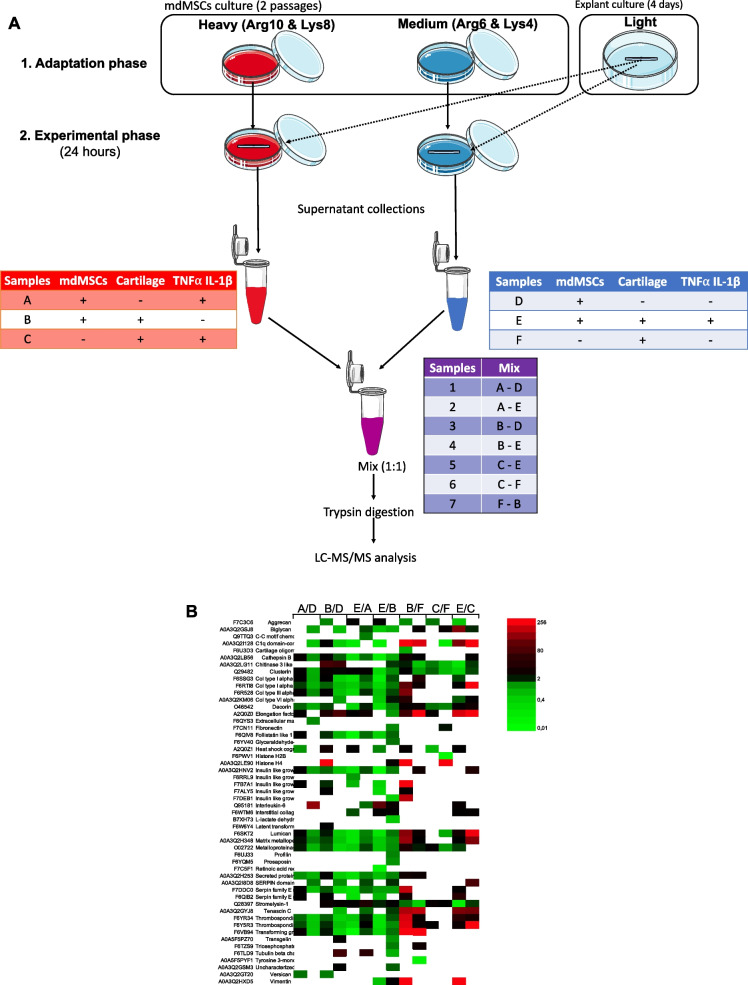
Secreted proteins identified by mass spectrometry with SILAC method. (**A**) Experimental design of SILAC experiment. Cells were first cultured with stable amino acids during 2 passages to let their incorporation into the cell proteome. After this adaptation phase, the experimental phase consists in a 24 h-exposure of mdMSCs in the presence or absence of pro-inflammatory cytokines and/or plugs of cartilage. After 24 h, supernatants were harvested, proteins extracted and mixed in a 1:1 ratio (w:w based on protein content) before processing for protein digestion and mass spectrometry analysis. The experiment was repeated at the next passage (reverse) and experimental conditions were switched between medium and heavy-labeled cells to avoid possible labeling bias. (**B**) Heatmap view of equine labeled proteins sequenced from 7 protein mixtures analyzed. Proteins sequenced for the comparison between 2 experimental conditions and for each replicate (forward and reverse) are listed on the left. Values are expressed as fold change related to the reference condition. The experiment was performed on 3 independent biological replicates but the identification in at least 2 donors was required to consider the protein in this analysis

The experimental phase was then conducted to analyze the proteome of mdMSCs in the presence of pro-inflammatory cytokines and/or plugs of cartilage without priming, as explained in the method section and schematized in Fig. 5A. Mass spectrometry analyses of the supernatants corresponding to the different conditions led to the sequencing of 52 different equine proteins in total, summarized in Fig. 5B. However, despite SILAC is considered as a gold standard quantitative proteomic method, we used this metabolic labeling only in a qualitative way to discriminate Equus proteins from Bos proteins. Indeed, in addition to a high variability between donors of the two cell types (mdMSCs and plugs of cartilage, see Table 4 of additional file 1 in Supplementary information for more details), we observed a poor reproducibility between forward and reverse replicates. Such a technical bias is not described in the literature, but it is the first time that a SILAC analysis is conducted mainly to ignore the bulk of contaminating FBS proteins. The relatively low amount of proteins of interest when compared to the large quantity of serum proteins might be responsible for this high variability between the quantitative ratios (Fig. 5 in Supplementary Material). Therefore, we only considered the identification of equine proteins but not the quantitative aspect.

As this set of data could not be used in a quantitative way, we decided to focus on 3 proteins with putative biological interest in the context of OA that were particularly highly sequenced in several experimental conditions: clusterin, decorin and MMP3 (also called stromelysin-1). Clusterin is a glycoprotein of cartilage extra-cellular matrix (ECM) especially implicated in immune response [53]. Decorin is a proteoglycan protein composing the ECM of cartilage [54], described to be secreted by MSCs and to have anti-inflammatory and anti-fibrotic properties [55]. MMP3 is a protease involved in catabolic function of the joint. This enzyme has different targets/substrates such as collagens II, III, IV, IX, X and XI [56].

**Fig. 6:**
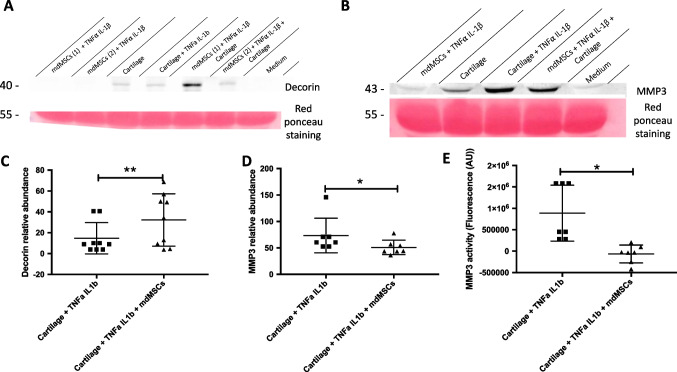
Effect of mdMSCs co-culture on proteins secreted by the cartilage plugs used as in vitro osteoarthritis model. (**A, B**) Relative abundance of decorin and MMP3 after 9 days of co-culture between mdMSCs and explants of cartilage in pro-inflammatory medium. mdMSCs [1] and [2] refer to cells from two different donors. The medium condition (containing medium + FBS) is a negative control. Red Ponceau staining was used as loading control. The quantification presented in (**C, D**) corresponds to the fluorescence signal intensity of decorin or MMP3 divided by the Red Ponceau staining optical density of the corresponding lane. Experiments were performed on 9 (decorin) and 7 (MMP3) biological replicates. (E) At the same time point, MMP3 activity in the supernatants was measured in the OA-like model co-cultured or not with mdMSCs (*n* = 7). *p*-values were calculated following Wilcoxon tests. * corresponds to *p*-value < 0.05; ** corresponds to *p*-value < 0.01

Western blot analyses were used to obtain semi-quantitative values for the abundance of those proteins between the different experimental conditions after 9 days of co-culture. The data concerning clusterin abundance was not exploitable as the antibody revealed a strong Bos contaminant protein in the negative control (composed of FCS-containing culture medium) (Additional file, Fig. 3). Western blot analysis of decorin abundance showed that while mdMSCs do not secrete decorin (the signal is comparable to the negative control containing only culture medium), this protein is spontaneously secreted by cartilage plugs. Pro-inflammatory cytokines strongly increase decorin secretion by cartilage. Most interestingly, decorin secretion by cartilage is significantly increased when plugs were cultured with mdMSCs (Fig. 6A and C). Regarding the catabolic enzyme MMP3, its abundance and activity were significantly decreased in the presence of mdMSCs (Fig. 6B, D and E).

## Discussion

The goal of this work was to determine whether mdMSCs could represent a valuable source for cell therapy in the context of joint disease like osteoarthritis.

Different in vitro models of OA exist in the literature based on the differentiation of mdMSCs into chondrocytes but this method was not convincing regarding the limited differentiation efficiency and/or high donor variability [57]. The culture of primary chondrocytes is another possibility to work with a phenotype closer to the physiological situation. However, culture conditions seem to rapidly lead to dedifferentiation [58]. Cartilage explants allow to work with chondrocytes embedded in their matrix, a favoring element to maintain the chondrocyte phenotype [59]. Explants of cartilage, mainly human and bovine tissues, used for in vitro articular part, were already studied by several teams showing especially that after approximately 2 weeks of culture, they were morphologically intact [60], collagen content remained stable [61] but chondrocytes death occurs at the edge of the explant [62]. Our results showed that after 15 days, chondrocytes mortality did not exceed 16% for equine cartilage explant conserved in culture condition (Fig. 1).

The first experiment consisted in priming explants of cartilage with pro-inflammatory culture media for 3 days, to orient the phenotype of chondrocytes toward “OA-like” phenotype. We choose a low concentration of pro-inflammatory cytokines when compared to other research groups because we wanted to mimic the chronic process of OA, for which TNFα and IL-1β concentrations are less than 1 ng/mL in the synovial fluid from arthritic human knees [63]. We showed that human cytokines induce NF-kB translocation of equine cells and expression of IL-6 and IL-8 genes (Fig. 2). After 3 days of exposure to this inflammatory cocktail, explants of cartilage release pro-inflammatory actors such as IL-6, GAG and NO_2_^−^. After 9 days, the abundance and activity of MMP3 were also increased in the supernatant of cartilage explants cultured with TNFα and IL-1β (Fig. 3). These culture conditions thus induce an inflammatory response from ex vivo cultured cartilage, mimicking an OA-like phenotype of equine cartilage, in line with a comparable phenotype observed in a similar human model [64]. These results were expected as genes encoding IL-6 [65], MMP3 [66] and inducible nitric oxide synthase (iNOS), the enzyme responsible for NO synthesis in chondrocytes [67], are NF-kB target genes. IL-6 is a cytokine with pleiotropic effects, which are mostly pro-inflammatory. On the cartilage, IL-6 contributes to decrease the production of type II collagen [68], activates proteoglycan catabolism [69] and increases the expression of MMPs [70], although IL-6 can also exert some cartilage protective effects such as the induction of TIMPs [71]. NO is another secreted compound considered as OA cartilage signature, as shown in animal models [72] and in cartilage obtained from human OA patients [73]. At the molecular level, NO can, in the presence of oxygen and reactive oxygen species, give rise to reactive components detrimental to macromolecules, but it can also regulate cell metabolism and cell signaling by S-nitrosylation of cysteine residues [74]. iNOS inhibitors are currently under development for OA therapy, as the inhibition of NO production was shown to reduce inflammatory markers [72, 75], to decrease MMP3 expression and to enhance ECM anabolic markers such as aggrecan and collagen type II [76].

After showing that this in vitro model is functional, mdMSCs were co-cultured with explants of “OA-like” cartilage, in pro-inflammatory media. mdMSCs were able to decrease the GAG release from explants after 4 and 9 days of co-culture, as already reported by other groups for human and murine cells (from adipose tissue and bone marrow, respectively) [77, 78], but did not affect the NO_2_^−^ production (Fig. 4). The absence of effect of mdMSCs on NO production is in contradiction with data obtained in a human explant-based model of OA induced by IL-1, where the conditioned medium of adipose-derived MSCs provoked a 20% decrease in NO production after 3 days of incubation [78]. This discrepancy between the two studies might be due to one of the many parameters at the origin of MSCs diversity, such as tissue of origin, method of isolation, degree of expansion [79], and/or to the species particularities.

As the beneficial effect of MSCs on joint suffering from osteoarthritis is mostly attributed to MSCs secreting bioactive factors [80], profiling the MSCs secreted proteins was required to characterize the paracrine potential of mdMSCs and define their in vitro therapeutic potential in OA. We worked with mass spectrometry-based untargeted proteomic approach to identify the most secreted proteins. As mdMSCs are cultured with 20% of FBS, a very high source of contaminating proteins, we have adapted the SILAC method to label secreted proteins from Equus mdMSCs cultured with 20% of FBS in the in vitro model of OA. We combined SILAC method with ion mobility and PASEF mass spectrometry technology to improve sensitivity and specificity. Trapped Ion Mobility Spectrometry (TIMS) adds a dimension of separation necessary to resolve the additional complexity of the samples analyzed by the SILAC method.

Supernatants of mdMSCs cultured in conditions of the OA in vitro model were analyzed for 3 biological replicates. Although SILAC is a gold standard quantitative proteomic approach, we were not able to use this set of data in a quantitative way due to high variability between biological and technical replicates and forward/reverse experiments. A first explanation is that 90% of identified proteins were Bos (data not shown) due to the presence of FBS proteins. It was therefore difficult to accurately quantify the low abundance of equine secreted proteins in such a complex sample characterized by a very large dynamic range. In addition, the reproducibility between replicates (biological and technical) was relatively low. The biological variability can be attributed, at least partly, to the fact that different donors of mdMSCs and explants of cartilage were used (supplementary information, Table 4). Finally, each experiment was repeated in forward and reverse labeling (medium and heavy) at 2 consecutive passages, which probably further increased the poor reproducibility of obtained values.

Nevertheless, 52 Equus proteins were robustly identified (Fig. 5B). While the goal of our study was not to characterize the proteins secreted from cartilage explants, we identified 15 secreted proteins in samples prepared from cartilage explants (without any mdMSCs) either treated or not with the pro-inflammatory cytokines (Supplementary information, Table 5). These proteins were labeled with stable isotope and identified for Equus species. However, on the contrary to mdMSCs, cartilage explants were not labelled during a "SILAC incorporation phase" (see Materials and Methods section). The cartilage explants were only exposed to labelled amino acids during the 24 h-exposure to mdMSC co-culture, meaning that these 15 proteins were rapidly synthetized from labelled amino acids present in the medium, suggesting a rapid turnover. We can thus suspect that the proteins identified in Table 5 (additional file) are not representative of all secreted proteins from explant. Nevertheless, 11 proteins out of 15 are in accordance with osteoarthritis human chondrocyte secreted proteins [81] as synthesized in the last column of Table 5 (additional file). The mass spectrometry data thus supports the functionality of this in vitro OA model.

Proteins secreted by mdMSCs are also coherent with results from other research groups/laboratories. For example, the secretome of human MSCs exposed to pro-inflammatory condition for 24 h (with 25 ng/mL of IL-1β, 20 ng/mL of IL-6 and 25 ng/mL of TNFα) revealed several proteins (decorin, TIMP-1, MMP2, MMP3, cathepsin B, serpin E1, chitinase-3-like protein 1, collagen type III αI, follistatin like 1, tenascin, IGFBP4 and 6, IL-6, fibronectin and C–C motif chemokine 2) [32] that were also identified in this study related to horse secreted proteins of pro-inflammatory cytokines-stimulated mdMSCs. In addition, several actors related to transforming growth-factor-β (TGF-β) pathway were secreted by mdMSCs. TGF-β is described to have chondrogenic inductive ability and to stimulate type II collagen and aggrecan secretion [82]. Plasmin, MMP2, reactive oxygen species or thrombospondin-1 are molecules reported to cleave the interaction between TGF-β and its latency associated peptide allowing the binding of TGF-β to its receptor to induce this pathway activation [83].

As quantitative proteomic data could not be considered due to the lack of robustness, we performed western blot analyses and enzymatic activity assays on selected proteins of potential interest in the OA and identified in the co-culture medium (Fig. 6). MMP3 was selected as it is considered as a major actor in OA-induced degradation of the various components of the cartilage matrix. This secreted enzyme degrades collagen type II, IV, IX, X and proteoglycans and it can also activate other MMPs such as MMP-1 or MMP7 (reviewed in Mehana et al., [84]). In addition, studies conducted in MMP3 KO mice have confirmed the key role of this enzyme in the pathology as OA is much less pronounced in MMP3 KO mice, as compared to wild type animals [85]. The results showed that the 9-days co-culture of cartilage plugs with mdMSCs provokes a moderate but significant decrease in secreted MMP3 abundance, and a strong reduction in MMP3 activity measured in the supernatants (Fig. 6). This suggests that the protective effect of mdMSC is mostly exerted at the level of MMP3 activation and/or activity, rather than on gene induction. This is in line with the absence of modification in NO_2_^−^ production in the presence of mdMSCs (Fig. 4), as NO has been shown to contribute to MMP3 induction by IL-1β [76]. TIMP1, a powerful inhibitor of MMP3 and other MMPs [86], detected by the proteomic analysis of supernatants (Fig. 5), would be a strong candidate to explain the MMP3 activity decrease in the presence of mdMSCs.

Western blot analyses also highlighted the increase in decorin abundance in supernatants of cartilage plugs incubated with pro-inflammatory cytokines, an expected response as decorin promoter region is induced in response to a TNFα-stimulation [87]. But the most striking effect is the increase in the abundance of secreted decorin when OA plugs are incubated with mdMSCs, although decorin is not detected in mdMSCs cultured without plugs, despite the presence of TNFα and IL-1β. Anti-inflammatory properties of secreted decorin [55] could participate to reduce the catabolic effect of cartilage explants in pro-inflammatory media. The use of MMP3 inhibitors and anti-decorin neutralizing antibodies in this mdMSC-OA like explant model could confirm the catabolic effect of MMP3 and anti-catabolic properties of decorin. These results could validate the interest of these two candidate proteins as potential therapeutic targets already studied by Guarise and collaborators [88].

Based on the data generated in this study, we can propose the following model (Graphical abstract): TNFα and IL-1β are pro-inflammatory cytokines turning on the inflammatory phenotype observed in the in vitro model of OA. Chondrocytes respond to such inflammatory conditions by secreting IL-6 and activated MMP3, as well as by producing NO, leading to the degradation of the ECM of cartilage explant as measured by the GAG release. mdMSCs in this in vitro model of OA are able to release some anti-catabolic molecules such as TIMP1, a MMP3 inhibitor, and decorin, a protein known for its anti-inflammatory properties [54]. In this equine explant-based OA model, the secretion of these molecular actors/effectors, among others, might explain, at least partly, the therapeutic potential of mdMSCs in an inflammatory environment, mainly achieved by paracrine-dependent mechanisms.

## Conclusion

Our results led to characterize mdMSCs secreted proteins in an in vitro equine model of OA, in the presence of FBS. First, we have studied functional response of OA-like plugs of cartilage co-cultured with adherent mdMSCs and we have shown that these cells are able to act on cartilage by reducing the GAG release but not the NO_2_^−^ production. Then, we have identified 52 secreted equine proteins and more particularly 3 of them highly sequenced in several experimental conditions of this model. Finally, western blot analyses showed an increased abundance in decorin secretion and a reduced MMP3 activity and abundance in supernatants, which might be responsible for the anti-catabolic effect of mdMSCs on cartilage explants in pro-inflammatory conditions. According to mdMSCs anti-catabolic effect, this source of MSCs could be considered for the development of therapeutic product for OA. This proof of concept of mdMSCs therapeutic property needs to be validated in more complex in vitro models (containing especially synovial membrane) before applications could be translated in preclinical trials.

## Supplementary Information

Below is the link to the electronic supplementary material.

Supplementary file1 (DOCX 30 KB)

Supplementary file2 (DOCX 1432 KB)

## Data Availability

Data and analyzed are available from the corresponding author on reasonable request. The mass spectrometry proteomics data have been deposited to the ProteomeXchange Consortium via the PRIDE [89] partner repository with the dataset identifier PXD031924 and 10.6019/PXD031924.

## Abbreviations

*DMEM*: Dulbecco’s modified Eagle’s medium
*ECM*: Extra-cellular matrix
*FBS*: Fetal bovine serum
*GAG*: Glycosaminoglycans
*IL*: Interleukin
*md*: Muscle derived
*MMP*: Matrix Metalloproteinase
*MS*: Mass spectrometry
*MSCs*: Mesenchymal stem cells
*NO*_*2*_^−^: Nitrite
*OA*: Osteoarthritis
*PASEF*: Parallel accumulation serial fragmentation
*PTM*: Post-translational modifications
*RT*: Room temperature
*SDS*: Sodium dodecyl sulfate
*SERPIN*: Serine proteinase inhibitors
*SILAC*: Stable isotope-labeling by amino acids in cells culture
*TBP*: TATA binding protein
*TGF*: Transforming growth factor
*TIMP*: Tissue inhibitor of metalloproteinases
*TIMS*: Trapped ion mobility spectrometry
*TNF*: Tumor necrosis factor
