# Supplementary material for: Characterization of the Proteins Secreted by Equine Muscle-Derived Mesenchymal Stem Cells Exposed to Cartilage Explants in Osteoarthritis Model
Source: Stem Cell Rev Rep. 2022 Oct 22;19(2):550–67. doi: 10.1007/s12015-022-10463-4 (PMC9902419; doi:10.1007/s12015-022-10463-4)
Supplement: Supplementary file 1 — Supplementary file1 (DOCX 30 KB) [file 12015_2022_10463_MOESM1_ESM.docx]

**Characterization of the proteins secreted by equine muscle-derived mesenchymal stem cells exposed to cartilage explants in osteoarthritis model.**

Lola Dechêne^1, 2^, Margaux Colin^3^, Catherine Demazy^1,4^, Maude Fransolet^1,4^, Ariane Niesten^5^, Thierry Arnould^1^, Didier Serteyn^2^, Marc Dieu^1,4^, Patricia Renard^1,4,*^

- 1. Unité de Recherche en Biologie Cellulaire (URBC) - Namur Research Institute for Life Sciences (Narilis), University of Namur (UNamur), 5000 Namur, Belgium
  2. Department of Clinical Sciences, Anesthesiology and Equine Surgery, Faculty of Veterinary Medicine, B41, University of Liege, Sart Tilman, 4000 Liège, Belgium
  3. Department of Pharmacotherapy and Pharmaceuticals, Faculty of Pharmacy, Université libre de Bruxelles (ULB), 1050 Brussels, Belgium
  4. Mass Spectrometry platform (MaSUN) - Namur Research Institute for Life Sciences (Narilis), University of Namur (UNamur), 5000 Namur, Belgium
  5. Centre of Oxygen, Research and Development (CORD), Institute of Chemistry B6a, University of Liege (ULiège), Sart Tilman, 4000 Liège, Belgium

**Corresponding author:** Patricia Renard [patsy.renard@unamur.be](mailto:patsy.renard@unamur.be) URBC, UNamur, Rue de Bruxelles 61, 5000 Namur, Belgium

Table 1: Primers used for qPCR

| **Gene** | **Forward** | **Reverse** | **Efficiency** | **Source** |
| --- | --- | --- | --- | --- |
| Enolase | GTGCAGCCAACTTCAGTGAA | CCAGCTTTCCCAATGGCATT | 97% | Personal |
| IL-6 | AACAGCAAGGAGGTACTGGCA | CAGGTCTCCTGATTGAACCCA | 95% | ﻿10.1089/scd.2016.0209 |
| IL-8 | ACAACGCGTTCTCCATGGTT | CAACCCTACACCAGATCCACA | 99% | Personal |

Table 2: Stable isotopes information.

| **Amino acids** | **Molecular Weight shift** | **Concentration**  **(mM)** | **Eurisotop references** | **Condition** |
| --- | --- | --- | --- | --- |
| 13C6 L-Arginine-HCl | +6 | 0,69 | CLM-2265-H-0,05 | medium |
| 13C6 15N4 L-Arginine-HCl | +10 |  | CNLM-539-H-0,05 | heavy |
| 4,4,5,5-D4 L-Lysine-2HCl | +4 | 0,8 | DLM-2640-0,1 | medium |
| 13C6 15N2 L-Lysine-2HCl | +8 |  | CNLM-291-H-0,05 | heavy |

Table 3: Incorporation of isotope-labelled amino acids.

|  | | **Technical replicates** | | **Average** |
| --- | --- | --- | --- | --- |
| **Donor 1** | Forward | 98% | 98% | 98% |
|  | Reverse | 96% | 99% | 98% |
| **Donor 2** | Forward | 92% |  | 92% |
|  | Reverse | 98% | 94% | 96% |
| **Donor 3** | Forward | 95% | 96% | 95% |
|  | Reverse | 98% | 92% | 95% |

*Incorporation rates were measured in 10 proteins and 413 peptides, for every donor at forward (after 2 passages) and reverse (after 3 passages) conditions and for each technical replicate.*

Table 4: Donor's information

| **Age at harvesting** | **Sex** | **Donor for** | **Reason of euthanized** |
| --- | --- | --- | --- |
| 5 | Female | mdMSCs |  |
| 6 | Female | mdMSCs |  |
| 10 | Female | mdMSCs |  |
| 12 | Female | mdMSCs |  |
| 13 | Female | mdMSCs |  |
| 14 | Female | mdMSCs |  |
| 16 | Female | mdMSCs |  |
| 22 | Female | mdMSCs |  |
| 10 | Male | mdMSCs |  |
| 22 | Male | mdMSCs |  |
| 27 | Male | mdMSCs |  |
| 21 | Female | Cartilage – SILAC experiment | Colic |
| 3 | Male | Cartilage – SILAC experiment | Joint wound |
| 14 | Male | Cartilage – SILAC experiment | Laminitis |
| 7 | Male | Cartilage – Functional experiments | Colic |
| 9 | Male | Cartilage – Functional experiments | Tetanus |
| 12 | Male | Cartilage – Functional experiments | Colic |
| 14 | Male | Cartilage – Functional experiments | Colic |
| 16 | Male | Cartilage – Functional experiments | Colic |
| 17 | Male | Cartilage – Functional experiments | Colic |

*Table 5: Secreted proteins from plugs of cartilage and their ratio for forward and reverse experiments.*

| **Proteins** | **Forward ratio** | **Reverse ratio** | **Review of Sanchez et al.**  (56) |
| --- | --- | --- | --- |
| Aggrecan | 1.22 |  | ↓ in OA |
| Biglycan |  | **9.06** | ↑ in OA |
| C1q domain-containing protein |  | 0.17 | / |
| Chitinase 3 like 1 | 1.16 | 0.6 | ↑ in OA, ↓ with IL-1β |
| Clusterin | 1.38 | **0.62** | ↓ in OA |
| Decorin | 1.24 | 1.08 | / |
| Elongation factor 1-alpha 1 | **7.54** |  |  |
| Fibronectin |  | **2.08** | Comparable levels between OA and normal chondrocytes |
| Heat shock cognate 71kDa protein | 0.63 |  | ↑ |
| Histone H2B |  | 0.04 |  |
| Histone H4 |  | **256** |  |
| Lumican |  | 0.74 | / |
| TIMP-1 | **5.19** | 0.88 | ↓ with IL-1β |
| Stromelysin-1 | **9.23** | **2.46** | ↑ in OA, ↑ with IL-1β |
| Thrombospondin 2 | **3.07** |  |  |

Ratios show the fold change between pro-inflammatory cytokine-treated explants and non-treated explants and appear in bold when the fold change is at least 1.5 times modified in treated condition. These secreted proteins from equine explants are compared with the secretome of human chondrocytes exposed to OA or IL-1β, reviewed by Sanchez and collaborators in the last column of the table.
