# Supplementary material for: Characterization of the Proteins Secreted by Equine Muscle-Derived Mesenchymal Stem Cells Exposed to Cartilage Explants in Osteoarthritis Model
Source: Stem Cell Rev Rep. 2022 Oct 22;19(2):550–67. doi: 10.1007/s12015-022-10463-4 (PMC9902419; doi:10.1007/s12015-022-10463-4)
Supplement: Supplementary file 2 — Supplementary file2 (DOCX 1432 KB) [file 12015_2022_10463_MOESM2_ESM.docx]

**Characterization of the proteins secreted by equine muscle-derived mesenchymal stem cells exposed to cartilage explants in osteoarthritis model.**

Lola Dechêne^1, 2^, Margaux Colin^3^, Catherine Demazy^1,4^, Maude Fransolet^1,4^, Ariane Niesten^5^, Thierry Arnould^1^, Didier Serteyn^2^, Marc Dieu^1,4^, Patricia Renard^1,4,*^

- 1. Unité de Recherche en Biologie Cellulaire (URBC) - Namur Research Institute for Life Sciences (Narilis), University of Namur (UNamur), 5000 Namur, Belgium
  2. Department of Clinical Sciences, Anesthesiology and Equine Surgery, Faculty of Veterinary Medicine, B41, University of Liege, Sart Tilman, 4000 Liège, Belgium
  3. Department of Pharmacotherapy and Pharmaceuticals, Faculty of Pharmacy, Université libre de Bruxelles (ULB), 1050 Brussels, Belgium
  4. Mass Spectrometry platform (MaSUN) - Namur Research Institute for Life Sciences (Narilis), University of Namur (UNamur), 5000 Namur, Belgium
  5. Centre of Oxygen, Research and Development (CORD), Institute of Chemistry B6a, University of Liege (ULiège), Sart Tilman, 4000 Liège, Belgium

**Corresponding author:** Patricia Renard [patsy.renard@unamur.be](mailto:patsy.renard@unamur.be) URBC, UNamur, Rue de Bruxelles 61, 5000 Namur, Belgium

Figure 1: Full length blots for analysis of NF-kB p65 subunit in nuclear fractions of non-treated or treated mdMSCs with TNFα (1 ng/mL) and IL-1β (0.1 ng/mL) for 1 hour. TBP is used as a loading control. Positive control is a nuclear faction of murine RAW cells treated with 20 ng/mL of LPS for 1 hour and previously characterized to assess the nuclear translocation of p65.

Figure 2: Relative quantification of nuclear p65 abundance normalized to TBP in nuclear fractions of non-treated or treated mdMSCs with TNFα (1 ng/mL) and IL-1β (0.1 ng/mL) for 1 hour. p65 was 4.3 (first replicate) and 2.8 (second replicate) time more abundant in nuclear fractions of mdMSCs treated with pro-inflammatory cytokines than non-treated mdMSCs. The blots are in the figure 1 of this section or in the figure 2 of the article.

Figure 3: Original western blot analysis for clusterin relative abundance analysis after 9 days of co-culture between mdMSCs and explants of cartilage in pro-inflammatory medium. The medium condition (containing medium + FBS) is a negative control. The signal in this negative control revealed a strong Bos contaminant protein.

Figure 4: Original blots for Decorin and MMP3 analysis. (A, C) Relative abundance of decorin and MMP3 after 9 days of co-culture between mdMSCs and explants of cartilage in pro-inflammatory medium. The medium condition (containing medium + FBS) is a negative control. (B, D) Loading is controlled by Red Ponceau staining.

Figure 5: Heatmap view of equine labeled proteins sequenced under all the experimental conditions. Quantitative values obtained for the comparison between 2 experimental conditions and for each replicate (forward and reverse) are indicated. Values are expressed as fold change related to the reference condition. The experiment was performed on 3 independent biological replicates but the identification in at least 2 donors was required to consider the protein in this analysis.
